# Artificial Intelligence for Objective Assessment of Acrobatic Movements: Applying Machine Learning for Identifying Tumbling Elements in Cheer Sports

**DOI:** 10.3390/s25072260

**Published:** 2025-04-03

**Authors:** Sophia Wesely, Ella Hofer, Robin Curth, Shyam Paryani, Nicole Mills, Olaf Ueberschär, Julia Westermayr

**Affiliations:** 1 Institute of Physical and Theoretical Chemistry, Faculty of Chemistry, Leipzig University, 04103 Leipzig, Germanyrobin.curth@uni-leipzig.de (R.C.); 2 Department of Engineering and Industrial Design, Magdeburg-Stendal University of Applied Sciences, 39110 Magdeburg, Germany; ella.hofer@stud.h2.de; 3 Center for Scalable Data Analytics and Artificial Intelligence Dresden/Leipzig, 04105 Leipzig, Germany; 4 Brooks College of Health, University of North Florida, Jacksonville, FL 32224, USA; s.paryani@unf.edu; 5 Athletics Department, University of North Florida, Jacksonville, FL 32224, USA; n.mills@unf.edu; 6 Institute for Applied Training Science, 04109 Leipzig, Germany

**Keywords:** inertial measurement unit, cheerleading, artificial intelligence, machine learning, acrobatic sports, motion capture, gymnastics

## Abstract

Over the past four decades, cheerleading evolved from a sideline activity at major sporting events into a professional, competitive sport with growing global popularity. Evaluating tumbling elements in cheerleading relies on both objective measures and subjective judgments, such as difficulty and execution quality. However, the complexity of tumbling—encompassing team synchronicity, ground interactions, choreography, and artistic expression—makes objective assessment challenging. Artificial intelligence (AI) revolutionised various scientific fields and industries through precise data-driven analyses, yet their application in acrobatic sports remains limited despite significant potential for enhancing performance evaluation and coaching. This study investigates the feasibility of using an AI-based approach with data from a single inertial measurement unit to accurately identify and objectively assess tumbling elements in standard cheerleading routines. A sample of 16 participants (13 females, 3 males) from a Division I collegiate cheerleading team wore a single inertial measurement unit at the dorsal pelvis. Over a 4-week seasonal preparation period, 1102 tumbling elements were recorded during regular practice sessions. Using triaxial accelerations and rotational speeds, various ML algorithms were employed to classify and evaluate the execution of tumbling manoeuvres. Our results indicate that certain machine learning models can effectively identify different tumbling elements with high accuracy despite inter-individual variability and data noise. These findings demonstrate the significant potential for integrating AI-driven assessments into cheerleading and other acrobatic sports in order to provide objective metrics that complement traditional judging methods.

## 1. Introduction

Over the past few decades, cheerleading evolved from the sidelines of major team sport events to a professional competitive sport of its own right. It is now one of the most popular sports in the USA, with more than 3 million participants annually, and increasing worldwide popularity [1,2]. On the modern competitive level, the choreographed routines of cheerleading combine elements from gymnastics-like tumbling, stunts and pyramid acrobatics, jumps, dancing, and cheering [2,3]. Judges’ scoring is based on several objective and subjective categories comprising the difficulty and quality of execution of standing and running tumbling, and individual, pair, or group stunts, crowd interaction, expression, and motion technique [3]. Although tumbling involves many gymnastic-like manoeuvres, its character and execution in cheerleading differ substantially due to the aspect of team synchronicity, different ground properties, and their alignment to overall choreography and artistic expression. Therefore, an objective assessment of the quality of the execution of tumbling elements may prove challenging to the judges in competition and coaches during exercise given the fast, parallel execution of several athletes and their integration into a choreographic composition. 

With the advent of artificial intelligence in several areas of research and aspects of daily life, e.g., voice assistants such as Siri, autonomous driving technologies, or diagnostics in medicine [4,5,6], the potential for AI to also assist in sports biomechanics and exercise science garnered considerable interest [7,8,9,10,11,12]. In the realm of sports science, AI and machine learning (ML), a subset of AI using statistical algorithms to learn from data [13], are being increasingly applied in biomechanical gait analysis, performance optimisation, injury prevention, and team tactics [9,14,15]. These technologies, often used in combination with wearables that monitor heart rate, sleep quality, or other daily activities, offer the capability to process and analyse large volumes of data with high precision, enabling more objective and consistent assessments compared to traditional methods [16]. Thus, AI and ML can be seen as an assistance to provide support for training by means of data analysis [17].

Despite these promising advancements in AI applications within sports, their utilisation in acrobatic disciplines such as cheerleading remains limited. One study focused on using ML in teaching cheerleading [18]. However, the inherent complexity of cheerleading tumbling, which involves dynamic movements, precise timing, and coordination among team members, presents both challenges and opportunities for AI-driven assessment. ML algorithms, particularly those that are adept at handling time series data from inertial measurement units (IMUs), offer a promising approach to objectively quantify and evaluate tumbling elements. By capturing detailed motion data, these algorithms may be capable of identifying patterns and nuances in athletes’ performances that may be difficult to discern through human observation alone [19].

However, the application of ML in cheerleading is not without its challenges. Due to the inherent dependence of each element’s execution on the individual athlete’s unique biomechanics and technique, there is a risk of the “Clever Hans” effect [20], where models learn to recognise specific individuals rather than the intended performance characteristics. This can lead to models that perform well on training data but fail to generalise to new athletes or different performance contexts. Overcoming the limitations associated with individual variability and ensuring model generalisability can significantly enhance the objectivity and precision of performance evaluations. Addressing this issue requires careful model training, validation, and the incorporation of diverse datasets to ensure that ML algorithms can reliably assess tumbling elements across a broad range of performers [21].

In spite of these challenges, the potential benefits of integrating ML into the assessment of cheerleading are substantial. In this study, we seek to bridge the gap between ML and cheerleading by providing initial insights into the possibilities of using ML to support the assessment of cheerleading and tumbling elements. In particular, we investigate the feasibility of an ML-based approach that uses data from a single IMU to accurately identify tumbling elements in cheerleading routines. Using triaxial accelerations and rotational velocities, different ML algorithms are employed to classify and evaluate the execution of tumbling manoeuvres. Our analysis focuses on optimising data processing techniques to improve ML prediction capabilities and ensure that the models can effectively handle the complexity of cheerleading performances. The results show that ML models can generalise and accurately predict tumbling elements, while generalising to athletes never seen by the model. This integration into cheerleading has the potential to provide objective metrics that complement traditional scoring methods, thereby improving both competition evaluations and training methods as predictions fail in cases where manoeuvres are not executed cleanly. Our results show that ML has the potential to not only support fairer and more consistent scoring in the future, but also provides detailed feedback for athletes and coaches facilitating targeted training and performance improvements.

## 2. Materials and Methods

### 2.1. Subjects

A total of 16 well-trained collegiate cheerleaders (13 females, 3 males) from the coed cheerleading team of the University of North Florida, Jacksonville, FL, USA, took part in this study. Their mean age was 19.9 ± 1.0 (18–22) years, with a mean stature of 1.65 ± 0.10 m (1.42–1.85 m) and a mean body mass of 65.3 ± 13.5 kg (43.1–94.3 kg). The cohort consisted of 6 female flyers and 15 mixed-sex bases (7 female, 3 male). All subjects reported to have been free of injury for at least one month prior to the measurement. The team’s highly experienced coach instructed each session and was present during the entire measurement time. The study was conducted in accordance with the Declaration of Helsinki, and approved by both the Ethics Committee of the Department of Engineering and Industrial Design of the Magdeburg-Stendal University of Applied Sciences (protocol code EKIWID-2025-02-001EH, date 7 February 2025) and the Institutional Review Board of the University of North Florida (protocol code IRB#1970666-2, date 18 January 2023). All participants gave written informed consent to their participation.

### 2.2. Tumbling Elements

**Figure 1:**
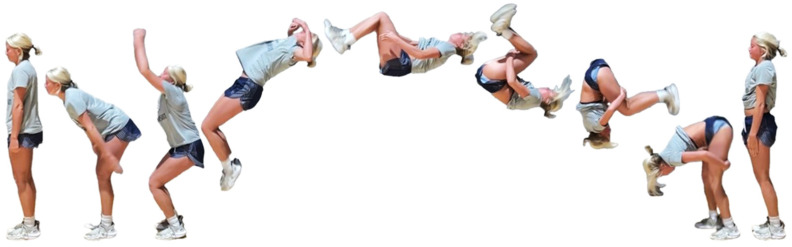
Movement sequence example (with stretched horizontal time axis) of a back tuck executed by one of the study’s cohort subjects. The IMU is worn under the pants approximately in the dorsal centre shorts waistband.

**Figure 2:**
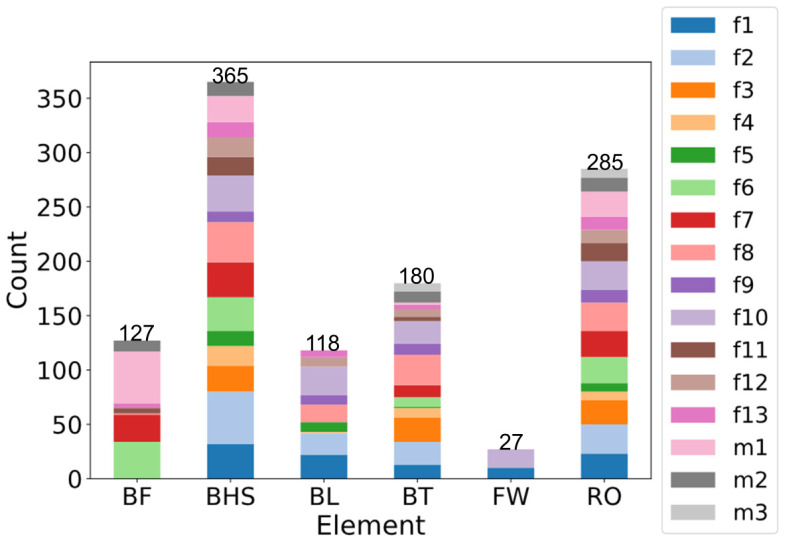
Distribution of tumbling elements performed by the 16 athletes (females f1–f13, males m1–m3) in the study. The six tumbling elements are back full (BF), back handspring (BHS), back layout (BL), back tuck (BT), front walkover (FW), and round off (RO). Numbers indicate the amount of data points sampled per tumbling element.

During the team’s three-month seasonal preparation period from September to November, a total of 1102 tumbling elements of six different types conducted by the 16 athletes were captured. These 1102 elements covered the following tumbling elements (Table 1): back full (BF, backflip with a full 360° twist), back handspring (BHS, the athlete jumps backwards into a reverse rotation about the transverse axis through a handstand position, blocks with the hands by shifting weight to the arms, and pushes off with the shoulders to land back on the feet to complete the turn); back layout (BL, backflip performed with a fully extended to slightly hollow position in the air), back tuck (BT, backflip performed in with the knees being pulled towards the chest while the hip is flexed, creating a tuck position in the air, Figure 1), front walkover (FW, tumbling element in which the athlete moves through a handstand into a split-legged bridge position and then stands up smoothly), and round off (RO, tumbling element in which the athlete supports the body weight with their arms while rotating sideways through an inverted position and landing on both feet on the ground at the same time, facing the direction from which they came) [3]. The distribution of these different tumbling elements is depicted in Figure 2. For each tumbling element, at least two different athletes performed the manoeuvre.

**Table 1:** Summary of tumbling elements included in data analysis.

Element	Abbr.	No.	Description [3]	Movement Sequence
Back full	BF	127	The back is a backflip (i.e., a 360° reverse rotation about the transverse axis) with a simultaneous full twist (i.e., a 360° rotation around the longitudinal axis).	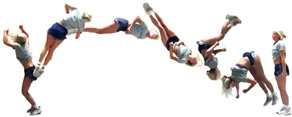
Back handspring	BHS	365	The back handspring is a reverse rotation about the transverse axis through a temporary handstand position.	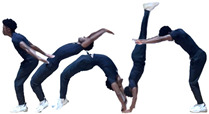
Back layout	BL	118	The back layout is a backflip (i.e., a 360° reverse rotation about the transverse axis) performed with a fully extended to slightly hollow position in the air.	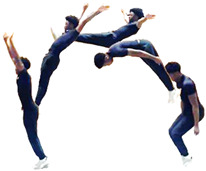
Back tuck	BT	180	The back tuck is a backflip (i.e., a 360° reverse rotation about the transverse axis) with the knees pulled towards the chest and the hips flexed mid-air (i.e., a tuck position).	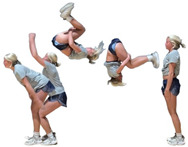
Front walkover	FW	27	The front walkover is an element in which the athlete moves through a handstand into a split-legged bridge position and then stands up smoothly.	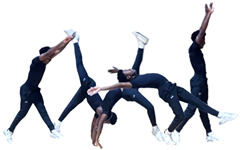
Round off	RO	285	The round off is an element in which the athlete supports the body weight with their arms while rotating sideways through an inverted position, landing on both feet on the ground at the same time, facing the direction from which they came.	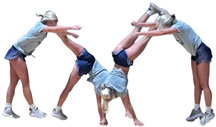

### 2.3. Data Acquisition

**Figure 3:**
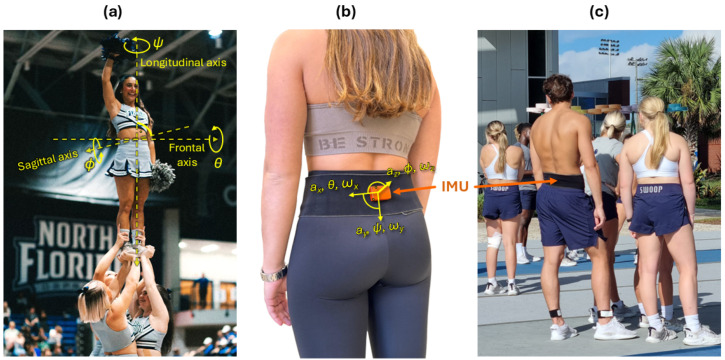
Utilised inertial measurement unit (IMU). (**a**) Anatomical axes and Euler angles: rotations about the athlete’s longitudinal axis are reflected in the angle *ψ*, while rotations about their frontal axis and sagittal axis relate to the angles *ϕ* and *θ*, respectively. (**b**) The Xsens MTw Awinda IMU (orange) is placed in the athlete’s lumbar region near the S1 vertebra using a comfortable, wide Velcro strap (black) put around the athlete’s waist. Accelerations *a_x_*, *a_y_*, and *a_z_*, as well as angular speeds *ω_x_*, *ω_y_*, and *ω_z_* are defined in the IMU’s local frame of reference as depicted. Note that the Euler angles roll (*ϕ*), pitch (*θ*), and yaw (*ψ*), however, are defined based on the athlete in the global frame of reference of the exercise hall (cf. a). (**c**) The rest of the Velcro strap is put on top of the IMU to further fix its position, as can be seen on the shirtless male athlete in the front.

Single wireless IMUs (Xsens MTw Awinda, Movella Inc., Enschede, The Netherlands) combining tri-axial accelerometers (tri-axial acceleration $\vec{a}={\left(a_{x},a_{y},a_{z}\right)}^{T}$, $\left|a_{x_{i}}\right|\leq 16\;g$), gyroscopes (tri-axial angular velocity $\vec{\omega }={\left(\omega_{x},\omega_{y},\omega_{z}\right)}^{T}$, $\left|\omega_{x_{i}}\right|\leq 2000{}^{\circ}\;s^{-1}$), and magnetometers (±800 μT) were placed in the athletes’ lumbar regions near the first sacral vertebra (S1) using Velcro straps (Figure 3) [22]. For particularly slim athletes, the IMU position was further secured by the tight-fitting shorts worn over it.

The internal data sampling rate was 1000 Hz for the accelerometers and gyroscopes, while the output data rate after sensor fusion was typically 120 Hz. In one session, due to a higher number of athletes, the data rate was reduced to 100 Hz [23]. The data were transmitted from the IMU to a receiving base station via a proprietary wireless protocol of the manufacturer using the 2.4 GHz band with a maximum free space range of 50 m. The actual maximum distance between the IMU and the base station that occurred during the motion capture sessions was less than 10 m. The IMU was initialised according to the manufacturer’s recommendations (i.e., proper temperature acclimatisation period to adapt to changed indoor or outdoor conditions, sensor self-initialisation in rest, sensor heading reset based on the ENU coordinate axis definition, etc.) before being attached to the athlete [23]. The type of IMU utilised has been widely used and validated for a wide range of motion analysis purposes in human sports science [22,23,24,25].

### 2.4. Computational Details

#### 2.4.1. Data Preprocessing and Unsupervised Learning Analysis

To make the data machine readable, the raw data had to be pre-processed. Therefore, each element that was performed less than 10 times was removed from the dataset in order to ensure a good split between training, validation, and test set for model training. In addition, the data had to be transformed such that each data point had the same input size. Each raw data point represents a time series of different length, due to the different duration of each measurement, and of dimensionality 9 (i.e., 9 input features characterising each movement, as explained in the previous section). Therefore, the data had to be reshaped. Two approaches were tested. First, the data were padded, i.e., zero values were attached to each of the 9 input vectors such that they had the same length. In a second approach, we tested linear interpolation as implemented in NumPy [26] to achieve a fixed length for each vector. Each data point then consisted of 9 vectors, each a length of 898, summing up to 8082 values per data point, representing one tumbling element performed by one athlete. These data were then normalised in each direction, concatenated, and reduced in their dimensionality for visualisation to obtain a first insight into how the data are clustered and what could be more suitable for ML algorithms. These data are referred to as “raw data”.

For dimensionality reduction, we performed principal component analysis (PCA) [27] in combination with k-means clustering [28] using scikit-learn [29]. The principal components plotted against each other for each data series, as well as their explained variance ratio, as discussed in Section 3. For comparison, the data were further transformed into a power spectrum via an autocorrelation function using fast Fourier transform (FFT) [30,31] and the Wiener–Khinchin theorem [32]. The data were further interpolated to 1000 data points per direction. We refer to these data as spectra data. The dataset was partitioned into a holdout test set containing 254 data points and a training set comprising 848 data points. To ensure proper learning and generalisation to athletes besides those in the training set, the holdout test set contained 4 athletes that were not included in the training set.

#### 2.4.2. Classification and Model Training

**Figure 4:**
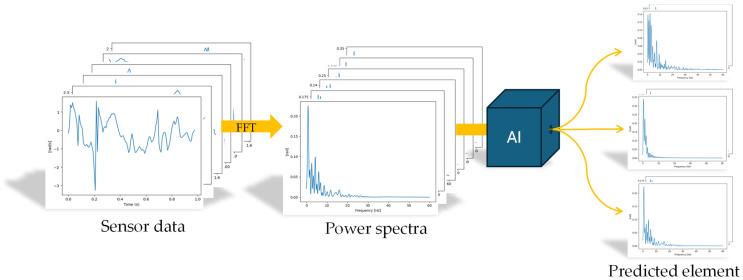
Data processing methodology showing raw data, spectra, and machine learning classification of three different elements (from top: BHS, BT, and RO).

To learn the relationship between the time series data and specific tumbling elements, supervised ML classification algorithms were employed [33]. These algorithms were trained to accurately map the input features to the corresponding tumbling elements. Gaussian process classification (GPC) [34,35], as implemented in the scikit-learn library [29], was selected for its robustness within small to medium-sized data and allowing for the prediction of an uncertainty measure next to each predicted class [36]. Training a GPC model involves learning the hyperparameters of the kernel function by maximising the marginal likelihood of the training data [34]. This process adjusts the kernel parameters to fit the data while balancing model complexity and overfitting [36]. Once trained, the model computes predictions for unseen data by conditioning the Gaussian process on the observed training data and applying Bayes’ rule [37]. The result is a predictive distribution for the latent function at each test point, which is then mapped to class probabilities [35]. Various GPC models were evaluated using different kernel combinations to determine the most effective configuration for classification tasks (see Table A1 in Appendix A). Among the tested kernels, the product of a constant kernel and a rational quadratic kernel [38] demonstrated superior performance, achieving the highest accuracy in identifying tumbling elements. The final method, including data preprocessing and ML prediction, can be seen in Figure 4.

**Figure 5:**
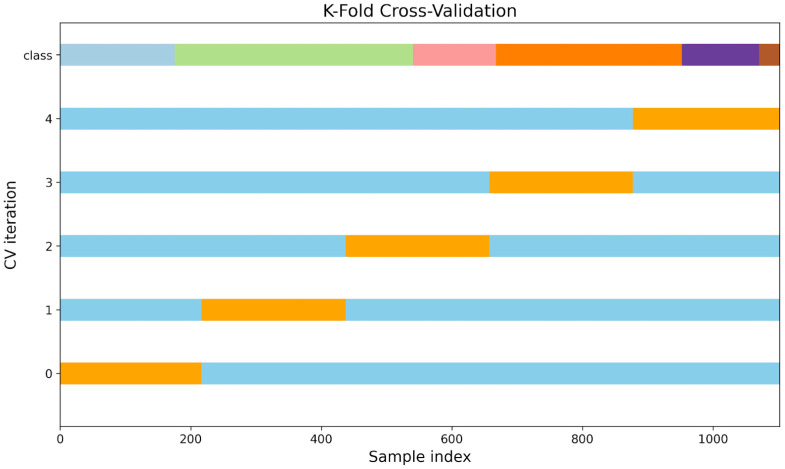
Schematic representation of *K*-fold cross-validation (CV) using five folds (*K* = 5). Top bar: class distribution (blue: BT, green: BHS, pink: BF, orange: RO, purple: BL, and brown: FW), and the lower 5 bars each represent a split between the training set (blue) and test set (orange) for one of the 5 folds used in CV.

**Figure A1:**
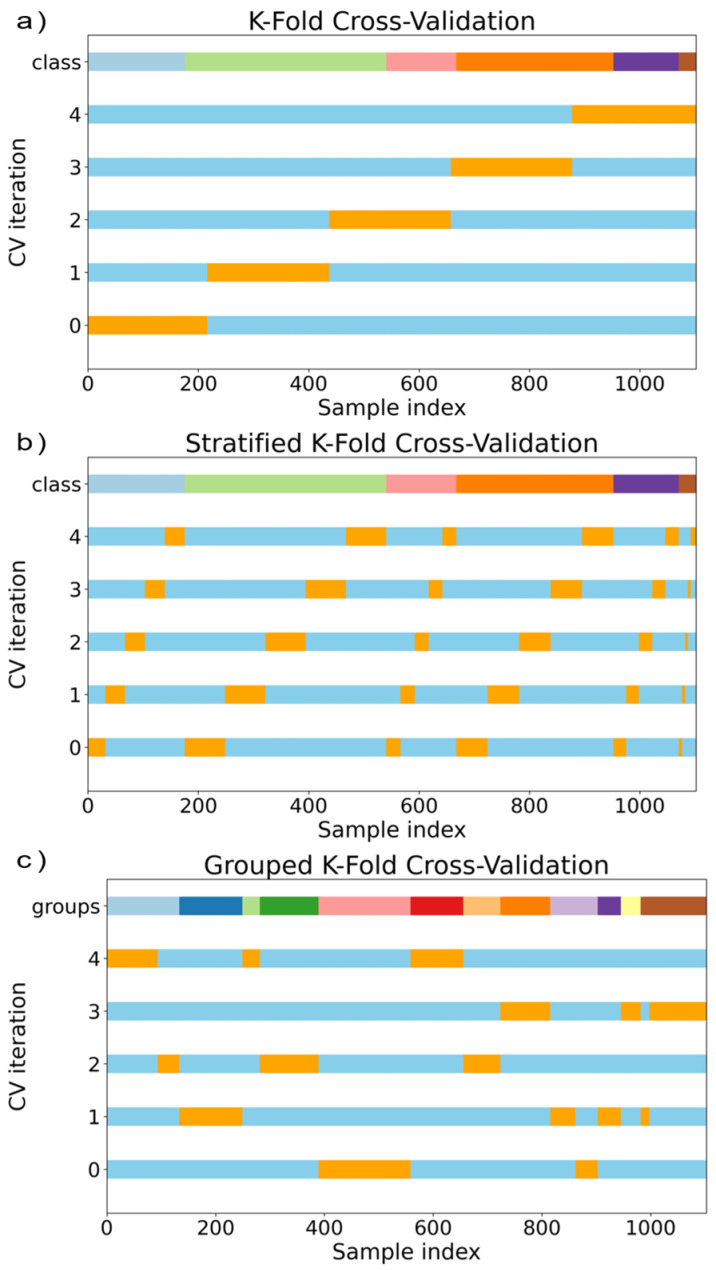
Schematic representation of cross-validation techniques used in this study. (**a**) Standard *K*-fold cross-validation, where the dataset is randomly split into *K* folds, with one-fold serving as the validation set (orange) and the remaining folds as the training set (blue). (**b**) Stratified *K*-fold cross-validation, which ensures the class distribution in each fold reflects the overall dataset. (**c**) Grouped *K*-fold cross-validation, where data points belonging to the same group (e.g., individual athletes) are kept together, ensuring no group appears in both the training and validation sets.

To ensure the reliability and generalisability of the models, a stratified [39] group *K*-fold cross-validation approach [40,41] with 5 folds was utilised next to a standard *K*-fold cross-validation approach. The *K*-fold cross-validation approach can be seen in Figure 5 with the stratified group *K*-fold approach visualised in Figure A1 in Appendix A. This method maintained the distribution of tumbling elements across each fold, thereby preserving the integrity of the evaluation process. Therefore, the dataset is split into training (blue) and validation (orange). In each round, the model is trained on the training set and evaluated against the validation set. The mean accuracy and standard deviation of the 5 models were calculated across the five folds to assess model performance comprehensively. Additionally, a randomised hyperparameter search [42] was conducted over 10 iterations to optimise the model parameters. The search aimed to identify the best-performing model based on the highest mean cross-validation accuracy. Once the optimal hyperparameters were determined, the best model was refitted using the entire training dataset and was tested against the holdout test set. The final model parameters are specified in Table 2.

To further understand the model’s ability to learn from the data, learning curves were generated to evaluate how the performance changes as the size of the training dataset increases [43]. Learning curves provide insight into whether the model is underfitting or overfitting and how effectively it generalises to unseen data. As the number of training samples increases, the model error on a holdout test set is expected to decrease and the accuracy is expected to increase. This behaviour can be examined by creating a learning-curve [43,44,45].

For this purpose, the training data were shuffled, and subsets of increasing size (100, 200, 400, and 600 entries) were extracted. For each subset, the model was refitted using (stratified group) *K*-fold cross-validation (5 folds), ensuring that the distribution of tumbling elements was maintained across all folds. After training on each subset, the model’s performance was evaluated by predicting the tumbling elements in the holdout test set. The scoring metric used was the mean accuracy of the model on the holdout set, averaged across the five folds, along with the standard deviation between the folds.

#### 2.4.3. Model Analysis

To better understand the contributions of individual features to the classification of tumbling elements, a feature importance analysis [46,47] was conducted. This analysis provides insight into which input features, such as triaxial accelerations or rotational speeds, are most influential in distinguishing between tumbling manoeuvres. Specifically, feature importance was evaluated by analysing the impact of perturbing or removing individual features on the model’s performance. This was achieved through a permutation importance approach [47], where feature values within the test set were permuted while keeping other features intact, and the corresponding decrease in model accuracy was measured [46,47,48].

## 3. Results

**Figure 6:**
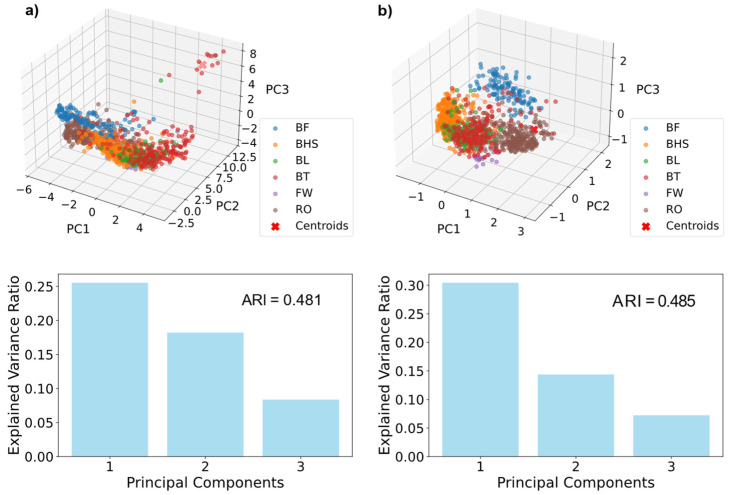
PCA plots with k-means clustering (**top**) and the explained variance ratio of these PCA plots as well as the adjusted rand index (ARI) for the clustering (**bottom**). (**a**) Results for padded spectra data and (**b**) interpolated spectra data.

The raw data were interpolated to the same size as each measurement was conducted for a different duration. Comparison of the PCA results (Figure 6), such as the adjusted rand index [49], showed that interpolation and padding of the data points both led to comparable results. However, the adjusted rand index of the interpolated data was slightly higher. Furthermore, GPC models trained on the interpolated data showed higher accuracies than models trained on padded data, prompting us to focus on interpolated data.

**Figure 7:**
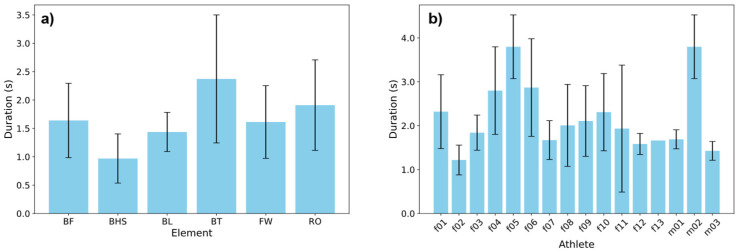
Comparison of mean durations of tumbling elements (error bars: SD). (**a**) Mean durations of the different elements over all repetitions. (**b**) Mean durations of the BT element performed by different athletes.

**Figure 8:**
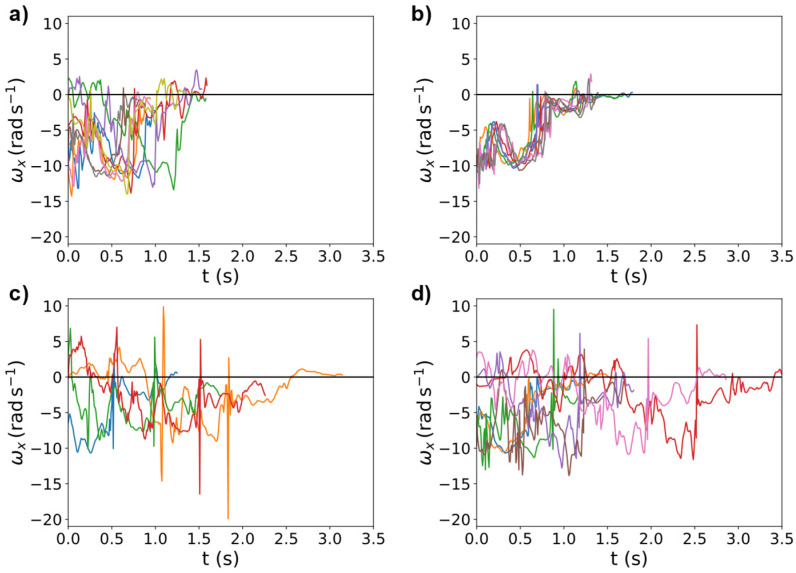
Raw $\omega_{x}$ data of four different athletes (depicted in (**a**–**d**)) performing BT. Colours represent different repetitions. The duration of this element varies among repetitions and athletes with an increasing maximal duration from (**a**–**d**).

The duration of the different tumbling elements showed high variance between the elements but also between different athletes performing the same element (Figure 7). Even when looking at the same element, this variance can be observed, making it necessary to extract important features to a time-independent scale (Figure 8). Therefore, we transferred the acceleration data into the frequency domain using the FFT of the autocorrelation function [30,31,32], ensuring the model learns the features themselves instead of recognising the length difference between exercises and increasing comparability between repetitions. This step was essential to extract meaningful frequency components from the data, such as dominant frequencies and spectral energy distributions, which are highly relevant for distinguishing between different tumbling elements.

The frequency domain representations of the data were then used as inputs for the ML model, i.e., GPC models. By leveraging the frequency domain features, the model could focus on periodic patterns and spectral characteristics that are not immediately apparent in the raw time domain signals. The GPC model, trained on these frequency domain features, was optimised to classify tumbling elements with high accuracy.

**Figure 9:**
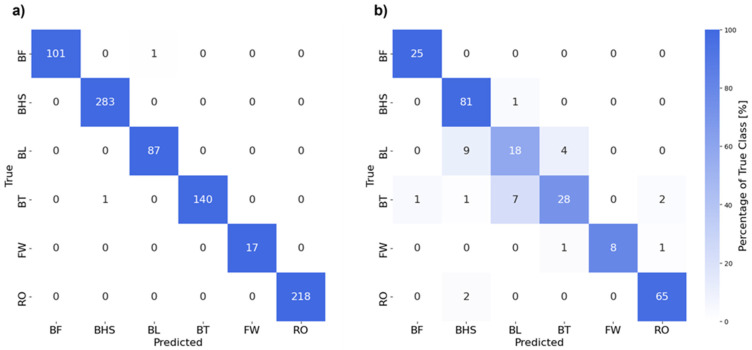
Confusion matrices showing correctly and wrongly predicted tumbling elements by the Gaussian process classification (GPC) model (**a**) on the training set and (**b**) the holdout test set. Abbreviations of tumbling elements are the same as in Figure 2.

Using this approach, we achieved an accuracy of 87.8% and 88.6% when using spectra data as input to GPC models with *K*-fold cross-validation and stratified group *K*-fold cross-validation, respectively. In comparison, using raw data without FFT led to an accuracy of ML models of 77.6% when using *K*-fold cross-validation and stratified group *K*-fold cross-validation. The resulting confusion matrix illustrates the amount of correctly predicted tumbling elements (diagonal) and wrongly predicted tumbling elements (off-diagonal). Figure 9a shows the performance of the whole training set and panel b on the holdout test set. In addition, Table 3 shows an additional performance measure for the holdout test set for each tumbling element.

**Figure 10:**
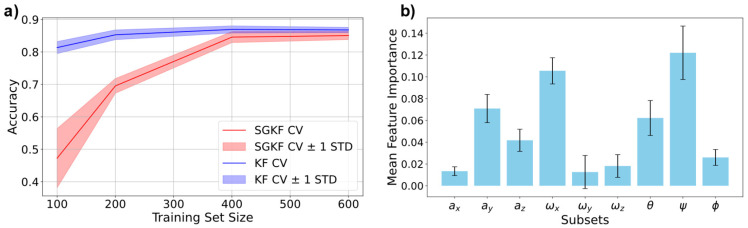
(**a**) Learning curves of the final Gaussian process classification (GPC) model using the *K*-fold (KF) cross-validation (CV) stratified group (SG) (KF CV) with 5 folds. The testing is conducted on a holdout test set containing athletes not included in the training and validation set. (**b**) Feature importance of the input features used for training the GPC model using 5-fold CV.

To ensure that models are learning properly, we further conducted learning curves (Figure 10a) and analysed the importance of the different features used as ML inputs (Figure 10b). The learning curves show how the model increases in accuracy with increasing dataset size, while feature importance analysis is crucial for interpreting model behaviour and identifying the most relevant biomechanical signals that drive the GPC model. A comparison of a learning curve using stratified group *K*-fold cross-validation (KF CV) is illustrated in Figure 10a for comparison.

**Figure 11:**
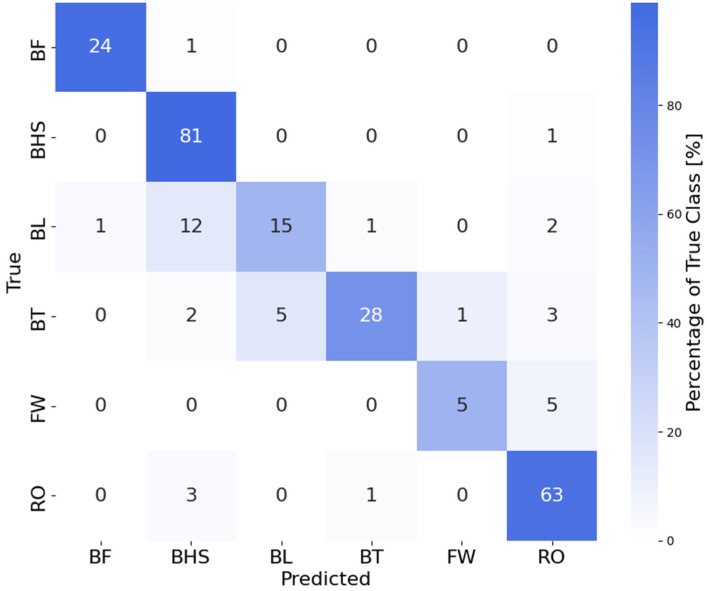
Confusion matrix showing correctly and wrongly predicted tumbling elements by the Gaussian process classification (GPC) model, trained on the three spectra with the highest feature importances only ($\omega_{x},\;a_{y},\phi$). Abbreviations of tumbling elements are the same as in Figure 2.

Finally, we performed training using only the three features with the highest importance identified by the analysis (Figure 10b) as input parameters for the GPC models. Results are shown in Figure 11 and Table 4. As can be seen, the performance of the GPC models is almost equivalent, although slightly worse, compared to the model that uses all features as input.

## 4. Discussion

### 4.1. Learning of the Classification Model

As illustrated in Section 3, the GPC model can predict tumbling elements with a high accuracy of approximately 90%. We performed analysis using classic *K*-fold cross-validation (KF CV in Figure 10a) and compared it to stratified group KF CV, which allowed a clear distribution of the different athletes and tumbling elements, ensuring that no data leakage was present. In KF CV, the dataset was randomly split into K folds, with each fold used as a validation set once while the remaining *K*-1 folds are used for training (Figure A1). However, in datasets where multiple samples originate from the same athlete (e.g., different tumbling elements performed by the same individual), this approach risks data leakage [50]. Specifically, data from the same athlete could appear in both the training and validation sets, allowing the model to learn individual-specific patterns rather than generalisable features, thereby overestimating performance. To address this, we employed SGKF CV (Figure A1b,c), which groups samples based on athletes, ensuring that no athlete’s data appeared in both the training and validation sets. This approach prevents data leakage and ensures that the model is evaluated on entirely unseen athletes, providing a more realistic estimate of its generalisation capability. Additionally, SGKF CV incorporates stratification to maintain the proportional distribution of tumbling elements across all folds, ensuring balanced representation of each class in both training and validation sets. Importantly, the CV is conducted to find ideal model hyperparameters. For testing accuracy (cf. values shown in the plot), we used a holdout test set that was not included in the training and validation set used for the learning curve. Both methods show similar results, leading to the conclusion that data leakage is not an issue here, further showing that the models do not show a “Clever Hans” effect [20] and can generalise well to unseen data.

For larger training set sizes (400 to 600 entries), both curves converge to similar accuracy values of approximately 0.9 with a reduced standard deviation, indicating improved model stability and generalisation as more training data are included. However, at smaller training set sizes, the SGKF CV curve (red) exhibits a much higher error and variance compared to the KF CV curve (blue). Specifically, the accuracy for SGKF CV drops significantly for a training size of 100, and the variance (±1 standard deviation) is also notably larger. This discrepancy is likely due to the stricter constraints imposed by SGKF CV, which ensures that entire groups (e.g., data from specific athletes) are kept separate between training and validation folds. In small datasets, this grouping constraint reduces the diversity of the training data, as fewer groups are available in each fold. Consequently, the model struggles to generalise effectively, leading to poor performance on the holdout set. As both methods led to similar results, further analysis is carried out using the 5-fold CV-trained model.

As already mentioned, feature importance analysis was carried out to identify the key features contributing to the classification of tumbling elements. However, it is important to note that the features used in this study are not entirely independent of one another. For example, angular speed about the *x* axis ($\omega_{x}=\dot{\theta }$) describes rotations about the frontal body axis (also referred to as horizonal, mediolateral or lateral axis). Analogously, $\omega_{y}=\dot{\psi }$ is primarily associated with rotations about the longitudinal (or vertical/craniocaudal) body axis, while $\omega_{z}=\dot{\phi }$ captures rotations about the sagittal (or anteroposterior/dorsoventral) body axis. Because individual anatomical conditions typically do not allow the IMU to be aligned with anatomical axes better than approximately ±5–10°, axial angular speeds may exhibit small contributions from the components of other axes. Nonetheless, the resulting uncertainty proves negligible for the given requirements for accuracy. Moreover, while the axial components of angular velocity are generally linearly independent, they are biomechanically related as they collectively describe the complex rotational dynamics of tumbling elements. Despite this interdependence, feature importance analysis remains a valuable tool. Permutation-based approaches account for the combined effects of features in the model by measuring the incremental impact of perturbing individual features while retaining their correlations with others [46]. In this context, feature importance reflects the relative contribution of each feature to the model’s decision-making process within the existing feature space. While $\omega_{x}$, $\omega_{y}$, and $\omega_{z}$ are related to pitch (θ), yaw ψ, and roll (ϕ) movements, respectively, their individual importance scores highlight the specific rotational axes that play the most significant roles in distinguishing tumbling elements in cheer sports.

While $\omega_{x}$, $\omega_{y}$, and $\omega_{z}$ are related to movements along the frontal, longitudinal, and sagittal anatomical axes, respectively, their individual importance scores highlight the specific rotational axes that play the most significant roles in distinguishing tumbling elements in cheer sports.

The results of the analysis, shown in Figure 10b, reveal that certain features consistently contributed more to the model’s performance. Specifically, rotational speed around the longitudinal and frontal axes ($\omega_{y}$ and $\omega_{x}$) exhibited the highest importance, underscoring their relevance in capturing the dynamic angular motions characteristic of tumbling manoeuvres. These rotational features reflect key biomechanical elements, such as body orientation and twisting motions, which are critical for differentiating between tumbling elements. Vertical acceleration of the centre of mass during ground contact (i.e., *a_y_*) also emerged as highly influential, highlighting its role in reflecting ground reaction forces and vertical displacements during tumbling. Conversely, sagittal rotations ($\omega_{z}$) and mediolateral accelerations (i.e., *a_z_*) exhibited lower importance, suggesting they were less critical for classification in this dataset. These results align with the biomechanical understanding that the tumbling elements studied are predominantly characterised by rotations about the longitudinal and frontal axes, while rotations about the sagittal body axis are usually part of a combined set of rotations, with the longitudinal and frontal axes being more decisive for characterisation. Similarly, mediolateral accelerations prior to flight phases are expected to play a subordinate role, as confirmed by our results. In essence, additional training of models on the three most important features only further support these conclusions.

Finally, as a kernel-based method, Gaussian process classification (GPC) has the advantage of effectively learning from limited dataset sizes, as demonstrated in the learning curve. Compared to neural network-based approaches (see Appendix A.4), GPC models perform better with smaller and less diverse datasets. However, as dataset size and diversity increase, neural networks may offer advantages. A key benefit of GPC is its ability to provide uncertainty estimates, which is particularly valuable when training on data that involve subjective judgments of movement execution. Future work will explore the potential of these models to support judging by incorporating evaluations from multiple individuals. Given the presence of noisy targets in such datasets, GPC’s uncertainty quantification could offer a significant advantage over models such as neural networks.

### 4.2. Model Analysis with Respect to Cheerleading Elements and Their Correlations

Finally, the confusion matrix shows the different elements that were predicted correctly (diagonal elements) within the test set and that were confused with each other (off-diagonal elements). The model demonstrated strong classification performance for BHS and RO, with 81 and 65 true positives, respectively, and minimal misclassifications. These elements have distinct biomechanical characteristics captured effectively by the model’s feature space. Similarly, the model showed reasonable performance for BT with 28 true positives, though there were some confusions with BL and RO, which share overlapping features in their execution.

For the BL class, the model achieved moderate accuracy with 18 true positives, but exhibited significant misclassifications. Specifically, BL was misclassified as BHS in nine instances and as BT in seven instances. This confusion is likely due to similarities in the rotational dynamics and most probably further exacerbated by inaccurate body position execution of the BL and BT elements by several individual athletes: both elements involve an aerial full 360° backward rotation about the athlete’s lateral axis without any ground contact in the flight phase [3]. The BHS, in contrast, is distinct by its short handstand support phase at a backward rotation angle around the lateral axis of approximately 180°. Although this unique acceleration impact peak of a BHS should be, in principle, easy to detect from IMU data, some practical limitations might come into play. We used a single IMU attached to the athlete’s lower back, which might not always be completely excluded from relative motion during vigorous movements. A certain degree of accelerometer measurement inaccuracy may thus be a factor. The confusion between BL and BT is most likely due to the similarity of the movement sequences, which differ only in the position of the legs in terms of the hip flexion angle [3], while the backflip rotations are similar (despite moderately differing moments of inertia about the lateral axis). The differentiation between BL and BT is further complicated when the execution of the BT is imprecise, as was observed in several athletes. A significant share of athletes tended to adopt a hip-flexed pike-like position rather than the correct, fully extended layout position. Similarly, distinguishing between BT and a back pike, even though not required in this study, may be even more challenging.

The FW class, with eight true positives, showed higher variability in predictions, likely due to its limited representation in the dataset. In the FW, there is a characteristic 360° forward rotation about the lateral axis of the athlete [3], which makes a confusion with a backflip element (i.e., BT and BL) unlikely. The FW has some characteristic kinematic similarities with an RO, but only up to about the middle of the first handstand phase. In contrast to the FW, the RO is then followed by an additional rotation around the body’s longitudinal axis [3], which is absent in the FW. In general, smaller class sizes, such as those for BF and FW, contributed to reduced performance for these elements, likely due to insufficient training samples. This imbalance can limit the model’s ability to generalise for underrepresented classes.

The analysis suggests several opportunities for improving classification performance. First, feature refinement could play a crucial role in enhancing separability between similar classes, such as BL and BT. Introducing advanced features, such as energy profiles, timing-based parameters, or transformations such as wavelet analysis, could help better differentiate overlapping movements. Second, data augmentation and balancing techniques could address the underrepresentation of certain classes, such as BF and FW, to improve the robustness of predictions. 

Nevertheless, the model shows that similar elements that were not conducted cleanly or executed correctly are more likely to be misclassified, leaving room for subjective interpretation and judging of elements. This observation highlights the potential of integrating judging criteria into the training process to better align the model’s classifications with human judgments of quality and execution. By incorporating subjective scoring metrics, such as deductions for improper form, synchronisation, or landing stability, future models could assist judges by providing objective and consistent metrics while leaving room for human interpretation in cases of ambiguity.

Such an integration would allow the model to move beyond mere classification of tumbling elements and assist in performance evaluation, potentially offering real-time feedback during competitions. For example, features related to execution quality could be trained alongside element classification, enabling the model to flag incomplete or incorrectly executed elements for further evaluation by judges. This could enhance the fairness and accuracy of scoring while reducing the cognitive load on human judges.

## 5. Conclusions

This study shows that machine learning can objectively classify tumbling elements in cheerleading using data from a single IMU. In total, 1102 recorded elements from 16 athletes, triaxial acceleration, and rotational speed data were transformed into the frequency domain for improved classification. Gaussian process classification achieved a high accuracy of approximately 90%, generalising well to unseen athletes even with a small amount of data. While challenges remain in distinguishing similar manoeuvres, these results highlight the potential of artificial intelligence to complement traditional judging. Future work should focus on refining feature extraction, expanding datasets, and integrating models that assess not only element classification, but also execution quality and technical precision, ultimately supporting more objective and consistent scoring in competition and training.

## Figures and Tables

**Table 2:** Summary of model parameters for different models found after randomised search.

Data Type	Split Method	Constant Value	α	Length Scale
Raw data	K-fold	2205.374	0.129	2.662
	Stratified group K-fold	9030.786	0.115	3.647
Power spectra	K-fold	925.599	23,618.319	22.788
	Stratified group K-fold	116.713	0.752	0.291

**Table 3:** Summary of the precision, recall and f1-scores of the GPC model on the holdout test set for each tumbling element.

Element	Precision	Recall	f1-Score
BF	0.96	1.00	0.98
BHS	0.87	0.99	0.93
BL	0.69	0.58	0.63
BT	0.85	0.72	0.78
FW	1.00	0.80	0.89
RO	0.96	0.97	0.96
Accuracy	0.89		

**Table 4:** Summary of the precision, recall, and f1-scores of the GPC model trained on the most important features ($\omega_{x},\;a_{y},\phi$) on the holdout test set for each tumbling element.

Element	Precision	Recall	f1-Score
BF	0.96	0.96	0.96
BHS	0.82	0.99	0.90
BL	0.75	0.48	0.59
BT	0.93	0.72	0.81
FW	0.83	0.50	0.63
RO	0.85	0.94	0.89
Accuracy	0.85		

**Figure A2:**
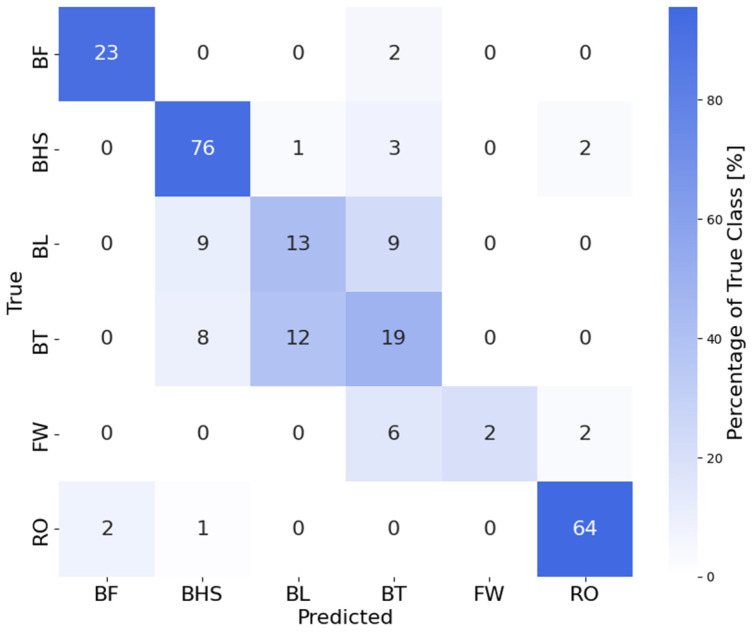
Confusion matrix showing correctly and wrongly predicted tumbling elements by the Gaussian Process Classification (GPC) model, trained on the raw time series sensor data. Abbreviations of tumbling elements are the same as in Figure 2.

**Figure A3:**
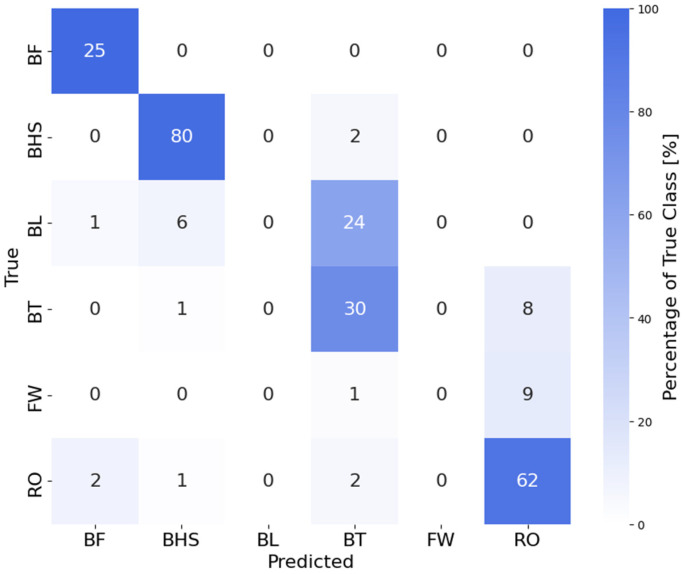
Confusion matrix showing correctly and wrongly predicted tumbling elements of the holdout test set by the neural network. Abbreviations of tumbling elements are the same as in Figure 2.

## Data Availability

Data will be available upon request and an example of a few data points (pseudonymised) is uploaded with the code. The code used to produce the results and train the ML models can be found on figshare: https://doi.org/10.6084/m9.figshare.28303298.v2.

## Appendix A. Machine Learning

### Appendix A.1. Cross-Validation of Classification Models

Figure A1a provides a schematic representation of the different cross-validation (CV) techniques used in this study to evaluate the performance of the Gaussian Process Classification (GPC) models. Cross-validation is a critical step in ensuring the reliability and generalisability of a model by partitioning the dataset into distinct training and validation sets. The methods illustrated here include standard *K*-fold cross-validation (a), stratified *K*-fold cross-validation (b), and grouped *K*-fold cross-validation (c), each tailored to address specific characteristics of the dataset and classification task. In this study, we used (a) and a combination of (b) + (c).

In standard *K*-fold cross-validation (Figure A1a), the dataset is split into K equally sized folds. Each fold is used once as the validation set (orange segments), while the remaining *K*-1 folds serve as the training set (blue segments). This method does not account for class distribution or group dependencies, making it suitable for datasets without structural or hierarchical groupings.

Stratified *K*-fold cross-validation (Figure A1a) ensures that each fold maintains the same proportion of class labels as the original dataset. This approach is especially useful for imbalanced datasets, where preserving class distributions during training and validation is crucial to avoid biased performance evaluations. The stratified sampling ensures that each fold is representative of the overall data distribution.

Grouped *K*-fold cross-validation (Figure A1c) addresses scenarios where individual data points are not independent but instead belong to identifiable groups (e.g., individual athletes or sequences in cheerleading routines). In this method, groups are kept intact, and no group appears in both training and validation sets simultaneously. This prevents data leakage and ensures that the model is evaluated on entirely unseen groups, reflecting its true generalisation ability.

### Appendix A.2. Different Kernel Performances

**Table A1:** Comparison of 6 different kernel combinations always consisting of a Constant Kernel C combined with a second kernel. Models were trained on spectral data with stratified group *K*-fold cross-validation during hyperparameter optimisation and evaluated on the holdout test set.

Kernel Combination	C × Matern	C × RBF	C × Rational Quadratic	C + Matern	C + RBF	C + Rational Quadratic
Test accuracy	88.50%	87.80%	88.60%	83.10%	84.30%	84.30%

### Appendix A.3. Kernel Performance on Raw Data

The raw time series data was normalised and padded to ensure consistent array shapes. A random hyperparameter search for a GPC model with a constant kernel multiplied by a Rational Quadratic kernel was conducted, using the stratified grouped 5-fold cross-validation methodology. The resulting confusion matrix and a performance-metrics summary table of the best GPC model on the holdout test set are shown in Figure A2 and Table A2, respectively.

**Table A2:** Summary of the precision, recall and f1-scores of the GPC model trained on the raw time series data on the holdout test set for each tumbling element.

Element	Precision	Recall	f1-Score
BF	0.92	0.92	0.92
BHS	0.81	0.93	0.86
BL	0.50	0.42	0.46
BT	0.49	0.49	0.49
FW	1.00	0.20	0.33
RO	0.94	0.96	0.95
Accuracy	0.78		

### Appendix A.4. Neural Network Approach

To compare the performance of a gaussian process classifier to another widely used ML-technique, we trained a neural network classifier on the spectral data. The neural network architecture consisted of two 1D-convolutional kernels followed by mean pooling and three linear dense layers and reLU activation functions. This model had a total number of 217,864 parameters. The model was trained using stratified group 5-fold cross-validation and tested on the holdout test set. Figure A3 and Table A3 summarise the results on the holdout test set.

**Table A3:** Summary of the precision, recall and f1-scores of the neural network on the holdout test set for each tumbling element.

Element	Precision	Recall	f1-Score
BF	0.89	1.00	0.94
BHS	0.91	0.98	0.94
BL	0.00	0.00	0.00
BT	0.51	0.77	0.61
FW	0.00	0.00	0.00
RO	0.78	0.93	0.85
Accuracy	0.78		
